# A *Drosophila* female pheromone elicits species-specific long-range attraction via an olfactory channel with dual specificity for sex and food

**DOI:** 10.1186/s12915-017-0427-x

**Published:** 2017-09-29

**Authors:** Sebastien Lebreton, Felipe Borrero-Echeverry, Francisco Gonzalez, Marit Solum, Erika A. Wallin, Erik Hedenström, Bill S. Hansson, Anna-Lena Gustavsson, Marie Bengtsson, Göran Birgersson, William B. Walker, Hany K. M. Dweck, Paul G. Becher, Peter Witzgall

**Affiliations:** 1 Department of Plant Protection Biology, Swedish University of Agricultural Sciences, Box 102, 23053 Alnarp, Sweden; 2 Biological Control Laboratory, Colombian Corporation of Agricultural Research, AA 240142 Las Palmas, Bogota, Colombia; 3 Department of Chemical Engineering, Mid Sweden University, Holmgatan 10, 85170 Sundsvall, Sweden; 4 Department of Evolutionary Neuroethology, Max Planck Institute for Chemical Ecology, Hans-Knoell-Strasse 8, 07745 Jena, Germany; 5 Chemical Biology Consortium Sweden, Science for Life Laboratory, Department of Medical Biochemistry and Biophysics, Karolinska Institutet, Scheeles väg 172, 17165 Solna, Sweden; 6 Department of Molecular, Cellular, and Developmental Biology, Yale University, New Haven, CT 06520 USA

**Keywords:** Olfaction, Sexual communication, Chemical ecology, Reproductive isolation

## Abstract

**Background:**

Mate finding and recognition in animals evolves during niche adaptation and involves social signals and habitat cues. *Drosophila melanogaster* and related species are known to be attracted to fermenting fruit for feeding and egg-laying, which poses the question of whether species-specific fly odours contribute to long-range premating communication.

**Results:**

We have discovered an olfactory channel in *D. melanogaster* with a dual affinity to sex and food odorants. Female flies release a pheromone, (*Z*)-4-undecenal (*Z*4-11Al), that elicits flight attraction in both sexes. Its biosynthetic precursor is the cuticular hydrocarbon (*Z,Z*)-7,11-heptacosadiene (7,11-HD), which is known to afford reproductive isolation between the sibling species *D. melanogaster* and *D. simulans* during courtship. Twin olfactory receptors, Or69aB and Or69aA, are tuned to *Z*4-11Al and food odorants, respectively. They are co-expressed in the same olfactory sensory neurons, and feed into a neural circuit mediating species-specific, long-range communication; however, the close relative *D. simulans*, which shares food resources with *D. melanogaster*, does not respond to *Z*4-11Al.

**Conclusion:**

The Or69aA and Or69aB isoforms have adopted dual olfactory traits. The underlying gene yields a collaboration between natural and sexual selection, which has the potential to drive speciation.

## Background

Sexual communication subserves mate-finding and ultimately reproduction, which relies on finding mates and food for offspring. Volatile pheromones transmit species-specific messages over a distance to facilitate mate finding, which is particularly adaptive in short-lived insects [1, 2]. Following mating, female insects search for larval food and oviposition sites, and both sexes forage to offset the nutritional cost of reproduction [3–6]. The search for mates and food is accordingly interconnected, and so is the response to sex and habitat olfactory signals. The sensory drive hypothesis reflects this interconnection and predicts that adaptation to natural habitats and food resources creates a sensory bias for sexual signals that match habitat features [7, 8].

Pheromones are released into an atmosphere that is filled with environmental, habitat-related odorants, some of which manifest mating sites and food sources. The response to sex pheromones and food or habitat olfactory cues (kairomones) falls under sexual and natural selection, respectively. Pheromones and kairomones are always perceived as an ensemble in a natural context, leading to an interaction of sexual and natural selection during adaptive divergence of sexual signalling, which is thought to facilitate premating reproductive isolation [9–12].

Olfactory sexual communication is studied at cellular and molecular resolution in the fruit fly *Drosophila melanogaster*, but volatile pheromones that elicit flight attraction and encode species-specific mate recognition have not been found. *Drosophila* is attracted to yeast and fruit odorants for feeding, mating and egg-laying [13–15] and the interconnection between perception of pheromones and food semiochemicals is a current research theme [4, 16]. For example, the male-produced sex pheromone cis-vaccenyl acetate (cVA) and food stimuli are integrated to coordinate feeding, courtship behaviour and oviposition site selection [17–20]. Perception of cVA is a current and outstanding paradigm for studying the molecular and neuronal logic of innate, olfactory-mediated reproductive behaviour [19, 21, 22]. However, cVA and other known olfactory pheromones are active only at close range, during courtship. In addition, they are all shared with other *Drosophila* species, which raises the question of whether they account for species-specific communication [23–26].

Interspecific matings between the sibling species *D. melanogaster* and *D. simulans* [27], or other closely related species, are inhibited by the female-produced cuticular hydrocarbon (*Z,Z*)-7,11-heptacosadiene (7,11-HD), which is perceived through gustatory receptors [28–31]. 7,11-HD conveys species specificity, but is not known to be perceived via an olfactory receptor (Or) and nor to elicit flight attraction. This led us to ask whether *Drosophila* uses, in addition, volatile pheromone signals that mediate specific mate recognition at a distance.

We have identified the first long-range, species-specific pheromone in *D. melanogaster*, which greatly expands our understanding of *Drosophila* social communication. A pair of Ors, feeding into the same neural circuit, has developed a dual affinity to this pheromone and to environmental semiochemicals, encoding adult and larval food. A blend of this pheromone and a food odorant specifically attracts *D. melanogaster*, but not the close relative *D. simulans*. This becomes an excellent paradigm for studying the interaction of social signals and habitat olfactory cues in premating reproductive isolation and phylogenetic divergence.

## Results

### *D. melanogaster* females produce a suite of volatile aldehydes

**Fig. 1:**
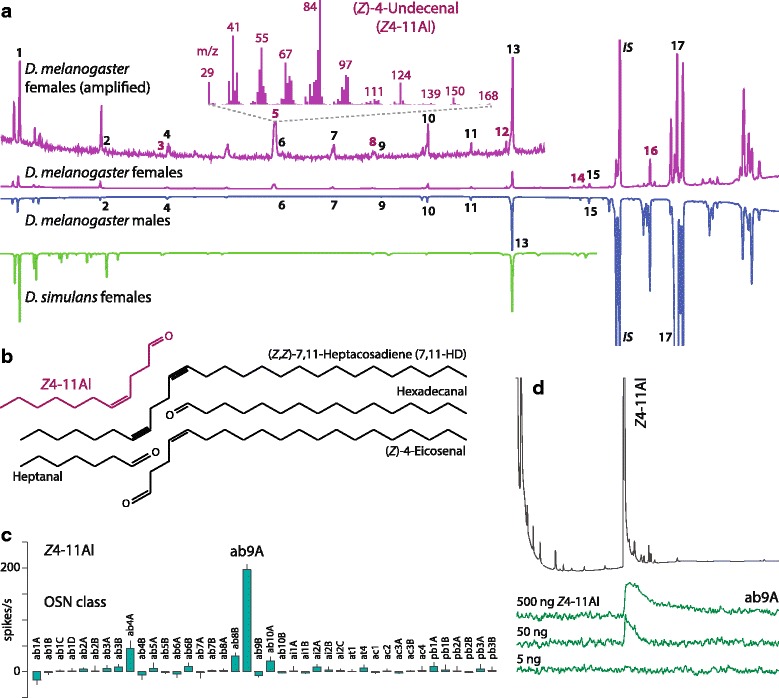
Chemical analysis of *Drosophila* headspace followed by electrophysiological screening of the candidate pheromone compound *Z*4-11Al. **a** Chromatograms of headspace collections from *D. melanogaster* (Dalby) females (lilac traces; upper trace: amplified signal; lower trace: entire chromatogram), males (blue trace), and *D. simulans* females (green trace). The headspace of *D. melanogaster* females contains 16 yet undescribed compounds: heptanal (**1**), octanal (**2**), (*Z*)-4-nonenal (**3**), nonanal (**4**), (*Z*)-4-undecenal (*Z*4-11Al) (**5**), undecanal (**6**), dodecanal (***7***), (*Z*)-4-tridecenal (**8**), tridecanal (**9**), tetradecanal (**10**), pentadecanal (**11**), (*Z*)-4-hexadecenal (**12**), hexadecanal (**13**), (*Z*)-4-octadecenal (**14**), octadecanal (**15**) and (*Z*)-4-eicosenal (**16**) (Table 1). Female-specific compounds are coloured, the most abundant cuticular hydrocarbon, 7-tricosene (**17**), is shown for reference, the internal standard (IS) was heptadecyl acetate. Inset: mass spectrum of the most abundant female-specific compound *Z*4-11Al. **b** Oxidation of the most abundant female cuticular hydrocarbon (*Z*,*Z*)-7,11-heptacosadiene (7,11-HD), affording two saturated and two unsaturated aldehydes, heptanal, hexadecanal, *Z*4-11Al and (*Z*)-4-eicosenal (Table 2). **c** Single sensillum recordings (SSR) from all *D. melanogaster* olfactory sensory neurons (OSNs) with *Z*4-11Al (error bars show SEM; n = 5). **d** SSR coupled to gas chromatography (GC-SSR), showing the response of ab9A to three different amounts of *Z*4-11Al

A focus in *Drosophila* pheromone research has been on cuticular hydrocarbons and cVA, which are active during close-range courtship [16, 24]. Our scope was to investigate volatile compounds mediating long-range communication, which are sensed by an Or at a distance and elicit upwind flight attraction. We therefore collected volatile compounds released by *D. melanogaster* flies in a glass aeration apparatus and found 16 aliphatic aldehydes, according to chemical analysis by gas chromatography-mass spectrometry (GC-MS). Males and females shared saturated aldehydes with a carbon chain length of C7 to C18, but mono-unsaturated aldehydes were released by females only (Fig. 1a; Table 1). The most abundant compound was identified as (*Z*)-4-undecenal (*Z*4-11Al) and synthesized.

**Table 1:** Saturated and unsaturated aldehydes found in headspace collections of *D. melanogaster* (Dalby) females and males

Compound	Females	Males
	(% ± SD, *n* = 5)^a,b^	(% ± SD, *n* = 5)^a,b^
Heptanal	3.1 ± 0.8	9.9 ± 4.8
Octanal	Traces	0.2 ± 0.5
Nonanal	4.1 ± 0.9	8.7 ± 7.9
(*Z*)-4-nonenal	Traces	–
Undecanal	Traces	–
(*Z*)-4-undecenal	23.3 ± 1.8	–
Dodecanal	7.9 ± 1.3	–
Tridecanal	Traces	–
(*Z*)-4-tridecenal	0.4 ± 0.9	–
Tetradecanal	11.2 ± 0.8	4.9 ± 4.1
Pentadecanal	3.2 ± 0.9	2.4 ± 2.1
Hexadecanal	27.8 ± 3.3	69.1 ± 9.7
(*Z*)-4-hexadecenal	2.9 ± 0.3	–
Octadecanal	4.7 ± 1.0	4.4 ± 3.9
(*Z*)-4-octadecenal	3.0 ± 0.6	–
(*Z*)-4-eicosenal	5.6 ± 1.7	–

^a^Headspace collection from batches of 20 flies

^b^Relative amounts

– Not found

GC-MS analysis showed that *Z*4-11Al was present also in cuticular extracts of females, albeit in lower amounts (0.27 ± 0.12 ng/female, *n* = 5) than in headspace collections (2.98 ± 0.81 ng/female, *n* = 5; *P* < 0.01 Mann–Whitney test). Cuticular profiles of *Drosophila* flies have been investigated, but *Z*4-11Al or other aldehydes have not been reported [23, 32, 33].

The sister species *D. simulans* did not release *Z*4-11Al, nor other monounsaturated aldehydes (Fig. 1a). Unlike *D. melanogaster*, *D. simulans* does not produce (*Z,Z*)-7,11-heptacosadiene (7,11-HD) [28, 29]. This led us to hypothesize that the production of mono-unsaturated aldehydes with a double bond in position 4 was linked to oxidation of di-unsaturated cuticular hydrocarbons. Oxidation of 7,11-HD is expected to generate two saturated aldehydes, heptanal and hexadecanal, and two unsaturated aldehydes, *Z*4-11Al and (*Z*)-4-eicosenal (Fig. 1b). This was experimentally verified by applying 100 ng of synthetic 7,11-HD to a glass vial (Table 2). Based on the cuticular hydrocarbon profile of *D. melanogaster* [32], 26 aldehydes are expected to be formed by oxidation, 16 of which were found in our headspace analysis (Table 1); others may have been below detection level. In hymenopterans, double bond-containing hydrocarbons have also been found to be oxidation precursors of aldehyde pheromones [34, 35].

**Table 2:** Autoxidation products of (*Z,Z*)-7,11-heptacosadiene (7,11-HD) eluted from a glass vial, 15 to min 75 min following application

Compounds^a^		15 min	30 min	45 min	60 min	75 min
	Molecular weight	(% ± SD, *n* = 3)				
Heptanal	114.2	–	–	–	–	–
(*Z*)-4-Undecenal	168.2	–	–	0.25 ± 0.35	0.92 ± 0.29	0.96 ± 0.18
Hexadecanal	240.2	1.22 ± 0.13	2.13 ± 0.52	2.92 ± 0.24	4.68 ± 0.38	5.09 ± 0.72
(*Z*)-4-Eicosenal	294.5	0.85 ± 0.16	1.61 ± 0.39	2.24 ± 0.1	4.00 ± 0.55	4.91 ± 1.27

^a^Predicted autoxidation products of (*Z,Z*)-7,11-heptacosadiene (7,11-HD) (see Fig. 1b)

– Not found

Next, single sensillum electrophysiological recordings (SSR) from all basiconic, trichoid, coeloconic, and intermediate olfactory sensilla in *D. melanogaster* (Fig. 1c) and gas chromatography-coupled SSR recordings (GC-SSR) from ab9 sensilla (Fig. 1d) showed that *Z*4-11Al strongly activates ab9A olfactory sensory neurons (OSN). A weaker response from ab4A neurons to *Z*4-11Al probably reflects the sensitivity of Or7a (expressed in ab4A) to aldehydes such as the leaf volatile (*E*)-2-hexenal [36–38].

### Or69aB responds to *Z*4-11Al

**Fig. 2:**
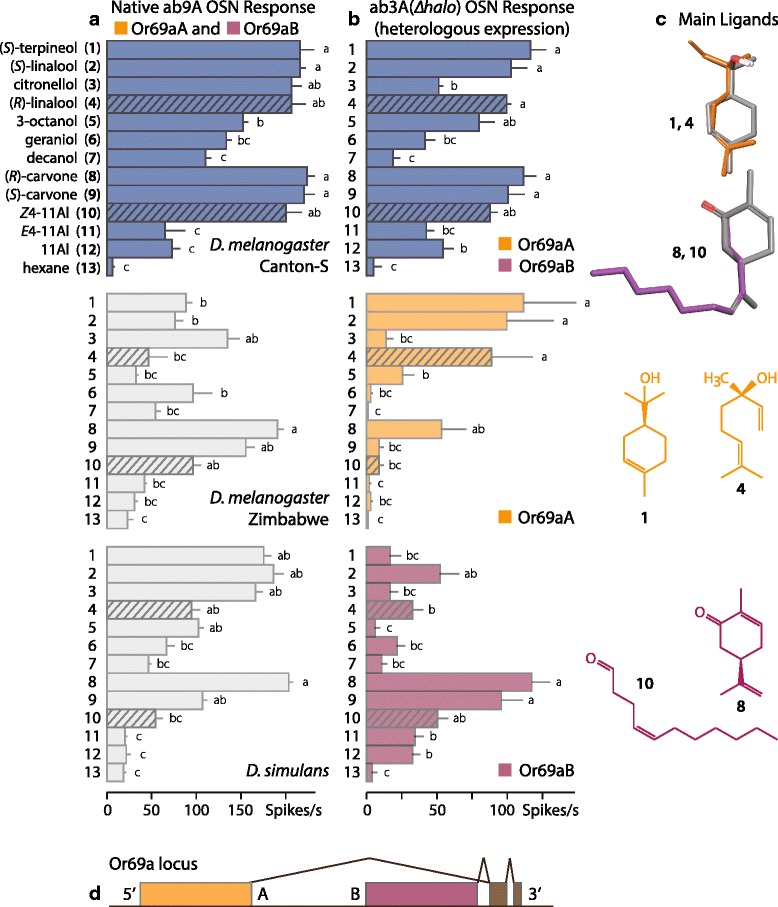
Single sensillum recordings (SSR) from neurons expressing Or69a variants. **a** SSR from ab9A olfactory sensory neurons (OSNs), in *D. melanogaster* (Canton-S, Zimbabwe) and *D. simulans* males, which natively express both variants Or69aA and Or69aB. **b** SSR from ab3A OSNs in *D. melanogaster*, heterologously expressing Or69aA and Or69aB, together and singly. (**a**, **b**) Test panel includes the known most active ligands for Or69a [39] and three aldehydes. Cross-hatched bars indicate compounds tested in the upwind flight assay (Fig. 3). Bars show mean responses and SEM (spikes/s), different letters indicate statistically significant differences for each fly type (*P* < 0.05; Mann–Whitney test, *n* = 5 for ab9A, *n* = 10 for ab3A). **c** Key ligands for Or69aA, (*S*)-α-terpineol (**1**) and (*R*)-linalool (**4**), and for or Or69aB, (*R*)-carvone (**8**) and Z4-11Al (**10**). Alignment of these ligands illustrates shared structural motifs. **d** Or69a locus, where coloured boxes A and B show unique exons and dark boxes show shared exons, generating the transcript variants Or69aA and Or69aB [40], which are co-expressed the same OSNs in ab9A sensilla in *D. melanogaster* [36, 40]

ab9A OSNs express Or69a [36]. We therefore screened ab9A OSNs with known ligands of Or69a [39] and *Z*4-11Al. In the *D. melanogaster* strains Canton-S and Zimbabwe, the monoterpene (*R*)-carvone elicited the strongest response from ab9A, although the response to *Z*4-11Al was not significantly different. In *D. simulans*, Z4-11Al elicited a significantly lower response than (*R*)-carvone (Fig. 2a).

The Or69a gene encodes two proteins, Or69aA and Or69aB (Fig. 2d), in most species of the *obscura* and *melanogaster* groups of *Drosophila* [40, 41]. Amino acids of Or proteins encoded by Or69aB and Or69aA transcripts are 44.5% identical, while amino acid identity between the Or69a variants and other *D. melanogaster* Ors is considerably lower, ranging from 7.0% to 22.8%. In *D. melanogaster*, the two isoforms are expressed together, in the same OSN population in ab9A sensilla [36]. Heterologous co-expression of both Or69a transcripts in ab3A (*∆halo*) empty neurons [42] produced a response similar to native ab9A OSNs, whereas individual expression revealed distinct response profiles for Or69aA and Or69aB (Fig. 2a,b). Or69aB responds best to both isomers of carvone, followed by *Z*4-11Al.

Carvone and *Z*4-11Al seem structurally different at first glance, yet they share a carbonyl functional group with an equidistant double bond in position 4 as the structural motif (Fig. 2b,c). Different ligands, upon binding to the same Or, are thought to adopt a complementary bioactive conformation. The strain energy required for any compound to assume a steric conformation that aligns with an active ligand should typically not exceed 5 kcal/mol [43]. Conformational analysis showed that *Z*4-11Al aligns with (*R*)-carvone, which elicited the strongest Or69aB response, at a strain energy cost of only 1.4 kcal/mol. Or69aA, on the other hand, is tuned to terpenoid alcohols and responded significantly less to *Z*4-11Al. The most active ligands (*S*)-α-terpineol and (*S*)- and (*R*)-linalool, which again share the functional group and a double bond in position 4, align at 3.0 kcal/mol (Fig. 2b,c). Conformational analysis substantiates that the most active ligands for Or69aA and Or69aB are structurally related.

### Z4-11Al elicits upwind flight attraction in *D. melanogaster*, not in *D. simulans*

**Fig. 3:**
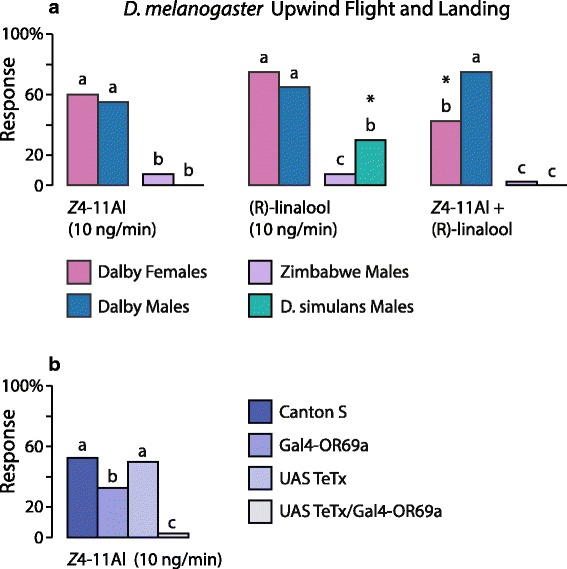
*Z*4-11Al mediates long range attraction and courtship in *D. melanogaster.* **a** Upwind flights to 10 ng/min of *Z*4-11Al and (*R*)-linalool, followed by landing at the source, in *D. melanogaster* (Dalby) males and females, *D. melanogaster* (Zimbabwe) males, and *D. simulans* males. Lower case letters indicate statistical differences between test insect strains and species, for each treatment. Asterisks indicate significant differences between treatments (*n* = 40, *P* < 0.001; binomial generalized linear models (GLMs) followed by post-hoc Wald pairwise comparison tests). **b** Upwind flights to 10 ng/min of *Z*4-11Al and (*R*)-linalool in *D. melanogaster* (Canton-S) males expressing a tetanus toxin in olfactory sensory neurons expressing Or69a, and in the parental lines. Letters indicate statistical differences within treatments (*n* = 40, *P* < 0.001; binomial GLM, followed by *Post-hoc* Wald pairwise comparison tests)

*Z*4-11Al elicited upwind flight and landing at the source in cosmopolitan Dalby and Canton-S strain *D. melanogaster* males and females. In contrast, males of the Zimbabwe strain and *D. simulans* were not attracted (Fig. 3a,b). This shows that *Z*4-11Al, in addition to its precursor 7,11-HD (Fig. 1), participates in sexual isolation between *D. melanogaster* and its sister species *D. simulans* [28, 29], and between cosmopolitan and African *D. melanogaster* strains [44–47].

Moreover, an admixture of *Z*4-11Al eliminated *D. simulans* attraction to the yeast volatile (*R*)-linalool [48] (Fig. 3a). *D. melanogaster* and its sister species *D. simulans* co-occur in the same habitat and use partially overlapping food resources. The very rarely occurring hybrid matings are sterile; the antagonistic interaction between pheromone and food stimulus might therefore be adaptive and point towards a contributing role of Or69a in reproductive isolation.

A tentative explanation for the significantly reduced attraction response of *D. melanogaster* females to the blend of *Z*4-11Al and linalool, compared to males (Fig. 3a), is that linalool both excites and inhibits other Ors, such as Or19a [14] and Or59b [49], and the interaction between these input channels with Or69a may be sexually dimorphic. Last, but not least, attraction to habitats involves many other odorants that may further modulate attraction to *Z*4-11Al.

The response magnitude of wild-type flies to Z4-11Al released at a rate of 10 ng/min (Fig. 3a) was similar to the upwind flight response to vinegar headspace, when the main compound acetic acid was released at microgram amounts per min [50]. This illustrates the high responsiveness and sensitivity of *D. melanogaster* males and females to Z4-11Al.

Finally, we used tetanus toxin (TeTx) transgenic fly lines to verify that OSNs expressing Or69a sense *Z*4-11Al. Upwind flight attraction was significantly reduced when Or69a OSNs were disrupted (Fig. 3b).

In summary, *Z*4-11Al is a powerful attractant that enables sexual communication and specific mate recognition at a distance. Its heterospecific role with respect to the sister species *D. simulans* was conserved in blends with the food attractant linalool.

## Discussion

We have identified a novel olfactory communication channel in *D. melanogaster*, wherein twin Ors expressed in the same OSN population simultaneously sense pheromone and food odorants. *Z*4-11Al is the first species-specific, long-range sex pheromone of *D. melanogaster*. It is produced by females and perceived by Or69aB in both sexes. The precursor of *Z*4-11Al is the cuticular hydrocarbon 7,11-HD (Table 2), which is known to mediate isolation between *D. melanogaster* and its sister species *D. simulans* during courtship [28–31].

*D. simulans* does not produce 7,11-HD [28, 29], and lack of attraction of *D. simulans* to *Z*4-11Al, as well as reduced attraction to a blend of linalool and *Z*4-11Al (Fig. 3a), suggests that *Z*4-11Al participates in sexual isolation between these two species. The aldehyde is far more volatile than 7,11-HD and suitable for long-range communication. African strains of *D. melanogaster*, especially from Zimbabwe, also show significant sexual isolation from cosmopolitan flies, which is due to chemosensory rather than visual and acoustic signals [44, 46]. The Zimbabwe strain of *D. melanogaster* produces mainly 5,9-heptacosadiene and only small amounts of 7,11-HD [45, 47], which explains why Zimbabwe males do not respond to *Z*4-11Al (Fig. 3a).

In addition to pheromones, Or69aB and its twin receptor Or69aA bind kairomonal terpenoids, such as linalool or terpineol, which are found in both fruit and yeast headspace. Citrus peel, a preferred oviposition substrate [14], and baker’s yeast, which elicits fly attraction and oviposition [13], are sources of all main ligands of Or69aA and Or69aB [48, 51]. Pheromone-releasing flies tint the pervading habitat and food odorants and thus shape and foreground a communication channel to facilitate mate finding. This is particularly adaptive when mating sites, such as fruit and berries, are abundant and widely spread. Social interactions affect the choice of food patches in *Drosophila* [52–54] and conspecific aggregations at feeding and egg-laying sites even promote the coexistence of species [55, 56]. Attraction of both females and males of *D. melanogaster* suggests a role of Z4-11Al in the formation of fly clusters at food sources.

Combined pheromone and food odour tuning by the two Or69a variants underscores the tie between sexual and natural selection during the evolution of specific mate communication, and is a convincing mechanism for the sensory drive hypothesis. Natural selection, in shaping sensory preference for the natural habitat and food sources, creates a sensory bias that will affect mate preference and the evolution of mate signals [7, 8].

Olfactory representations of other *D. melanogaster* Ors involved in food and pheromone perception project through separate channels to the lateral horn (LH) of the fly brain, where third-order neurons partially overlap and integrate [18, 19]. A cluster of LH neurons, termed P1, has been identified to collect olfactory and contact chemosensory signals and to elicit male courtship. Projection neurons from the DA1 glomerulus, responding to the male-produced aphrodisiac cVA, are one important source of input [19, 22, 24]. The question arises whether Or69a and projection neurons from the associated D glomerulus [36] contribute excitatory input to sexually dimorphic circuits in the LH. This could, for example, explain a sex-specific response to a blend of *Z*4-11Al and linalool (Fig. 3a), since linalool affords input also from other Ors [14, 49].

Most *D. melanogaster* OSNs express one Or, with the exception of ab10B neurons, which co-express Or49c and Or85f [57], and ab9A, which co-expresses Or69aA and Or69aB [36, 40]. Or69a is the first olfactory gene known to encode dual olfactory traits; Or69aA and Or69aB achieve a coordination of sex and food stimuli already in first order neurons, at the antennal periphery. This makes Or69a a prime target for selection, also because the tuning range of Ors evolves more rapidly than hardwired neural circuits in higher brain centres [58]. Differential tuning of Or69a in closely related cosmopolitan and African strains of *D. melanogaster* (Fig. 2a) corroborates this idea.

Gene duplication is thought to be a principal mechanism for Or repertoire expansion and evolution [59]. Since Or69aA and Or69aB are more similar to each other than to other Ors, it is conceivable that the Or69aA exon has arisen from a duplication of the ancestral Or69aB exon, which is found throughout *Drosophila* [41]. The twin receptors Or69aA and Or69aB facilitate adaptive changes in ligand tuning, without compromising the established functional role of the Or69a channel. Functional divergence has apparently been biased towards structurally related ligands (Fig. 2b,c) [39] and behaviourally and ecologically relevant odorant signals.

## Conclusion

Tuning changes in the two Or69a variants are constrained to a behavioural theme since they are expressed in one OSN and feed into a neural circuit mediating sex and food attraction. The two Or variants provide, on the other hand, degrees of freedom during adaptive divergence, since they allow fly populations to adopt new kairomone or pheromone signals; alteration of either one produces a new communication channel. Reproductive isolation may arise as a by-product [11, 60–62] and the Or69a gene therefore has the potential to drive speciation [63, 64]. Species in the *D. melanogaster* and *D. obscura* groups provide a rich substrate for studying Or ligand evolution and its consequences for disruptive selection on ecological interactions and mate choice.

## Methods

### Insects

Canton-S, Zimbabwe (S-29; Bloomington #60741) and Dalby-HL (Dalby, Sweden) [65] strains of *D. melanogaster* were used as wild-type flies for behavioural experiments. Canton-S was used for comparison with knockouts of the same background. Further tests were performed with the sister species *D. simulans*.

We used the Or69a-Gal4/UAS TeTx, tetanus toxin knockout line to verify the role of Or69a in flight attraction to *Z*4-11Al. Canton-S/UAS TeTx (Bloomington #28838 and 28997) and Canton-S/Or69a-Gal4 (Bloomington #10000) were used as parental controls.

Flies were reared on a standard sugar-yeast-cornmeal diet at room temperature (19–22 °C) under a 16:8-h light:dark photoperiod. Newly emerged flies were anesthetized under CO_2_ and sexed under a dissecting microscope. Virgin flies were identified by the presence of meconium, and were kept together with flies of the same sex. Flies were kept in 30-mL Plexiglas vials with fresh food. Experiments were performed with 3- to 5-day-old flies.

### Chemicals

*Z*4-11Al and (*E*)-4-undecenal were synthesized (see below). Commercially available compounds were (*R*)-carvone (97% chemical purity, CAS #6485-40-1, Firmenich), (*S*)-carvone (98%, CAS #2244-16-8, Firmenich), (*S*)-terpineol (97%, CAS #10482-56-1, Aldrich), (*S*)-linalool (97%, CAS #126–91–0, Firmenich), (*R*)-linalool (97%, CAS #126-90-9, Firmenich), citronellol (99%, CAS #106-22-9, Aldrich), geraniol (98%, CAS #106-24-1, Aldrich), 3-octanol (99%, CAS #589-98-0, Aldrich), decanol (99%, CAS #112-30-1, Fluka), and undecanal (99%, CAS #112-44-7, Aldrich).

### Chemical synthesis

Dry THF and dry Et_2_O were obtained from a solvent purification system (Activated alumina columns, Pure Solv PS-MD-5, Innovative technology, Newburyport, USA) and used in the reactions when dry conditions were needed. All other chemicals were used without purification. Reactions were performed under an Argon atmosphere unless otherwise stated. Flash chromatography was performed on straight-phase silica gel (Merck 60, 230–400 mesh, 0.040–0.063 mm, 10–50 g/g of product mixture) employing a gradient technique with an increasing concentration (0–100%) of distilled ethyl acetate in distilled cyclohexane. In cases of very polar products, chromatography was continued with ethanol in ethyl acetate (0–20%). Thin-layer chromatography was performed to monitor the progress of the reaction on silica gel plates (Merck 60, pre-coated aluminium foil), using ethyl acetate (40%) in cyclohexane as an eluent, and plates were developed by means of spraying with vanillin in sulfuric acid and heating at 120 °C. The purity of the product was checked with GC analysis on a Varian 3300 GC instrument equipped with a flame ionization detector (FID) using a capillary column Equity-5 (30 m × 0.25 mm id, d_f_ = 0.25 μm, with nitrogen (15 psi) as the carrier gas and a split ratio of 1:20). The oven temperature was programmed at 50 °C for 5 min followed by a gradual increase of 10 °C/min to reach a final temperature of 300 °C. An Agilent 7890 GC equipped with a polar capillary column FactorFOUR vf-23 ms (30 m × 0.25 mm i.d., d_f_ = 0.25 μm) was coupled to an Agilent 240 ion-trap MS detector for separation of some isomeric intermediates. The injector was operated in split mode (1:20) at 275 °C and a helium flow rate of 1 mL/min and a transfer line temperature of 280 °C. The analyses were performed in the external ionisation configuration. Electron ionisation spectra were recorded with a mass range of m/z 50–300 at fast scan rate. NMR spectra were recorded on a Bruker Avance 500 (500 MHz ^1^H, 125.8 MHz ^13^C) spectrometer using CDCl_3_ as solvent and internal standard.

#### (Z)-4-11Al

(*Z*)-4-11Al was synthesized via a modified version of Wube et al. [66] in 80% stereoisomeric purity. Esterification under acidic conditions with sulfuric acid in methanol resulted in 80% *Z*-isomer and a 93% yield over two steps. Stereoisomeric purity was controlled with NMR and GC-FID by comparing the analysis for acid and ester, the appearance of a small quartet, in the NMR spectra, at 1.96 indicates the presence of *E*-isomer. Gas chromatographic separation on a polar Varian factorFOUR vf-23ms of *Z*- and *E*-ester proved that the stereochemistry was not affected by the acidic conditions during esterification. Methyl (*Z*)-4-undecenoate was purified on regular silica gel and on silver nitrate impregnated silica gel to obtain a stereoisomeric purity of 98.6%. Methyl (*Z*)-4-undecenoate was reduced to (*Z*)-4-undecenol with lithium aluminium hydride in diethylether and oxidized to *Z*4-11Al with Dess-Martin periodinane in dichloromethane.

NaHMDS (6.78 mmol, 1 M in hexane) was added dropwise, over 30 min, to a suspension of (3-carboxypropyl)triphenylphosphonium bromide (1.45 g, 3.39 mmol) in THF (25 mL). The mixture was stirred for 2 h then cooled to 0 °C on an ice/water bath, and heptanal (0.387 g, 3.39 mmol) in THF (2.5 mL) was added slowly over 15 min. The mixture was stirred for 5 h at 0 °C then allowed to reach room temperature overnight. The reaction was quenched with H_2_O (20 mL) and the organic solvent was evaporated. The remaining water phase was extracted with Et_2_O (3 × 20 mL), the obtained organic phases discarded, and the basic aqueous phase was acidified with HCl (2 M) until pH 1 and extracted with Et_2_O (3 × 20 mL). The combined organic phases were dried over MgSO_4_ (anhydr.) and the solvent evaporated off. The obtained crude product was dissolved in pentane, cooled at –18 °C and filtered to remove the precipitated OPPh_3_ followed by evaporation of the solvent to result in 0.547 g of a yellow oil (87.5% yield). ^1^H-NMR: 5.52–5.30 (m, 2H), 2.35 (m, 4H), 2.04 (q, *J* = 6.5 Hz, 1.6H, *Z*-isomer), 1.96 (q, *J* = 6.5 Hz, 0.4H, *E*-isomer), 1.37–1.19 (m, 8H) and 0.89 (t, *J* = 7 Hz, 3H) ppm. The NMR data is in accordance with data previously reported [66, 67]. The relationship by integration between protons at 2.04 and 1.95 indicates approximately a *Z*:*E* ratio of 80:20, which is supported by GC-MS analysis on a Varian factorFOUR vf-23ms column. The obtained crude product was used in the next step without further purification.

#### Methyl (Z)-4-undecenoate

(*Z*)-4-11Al (0.547 g, 2.97 mmol), as synthesized above, was dissolved in methanol (15 mL) and seven drops of concentrated H_2_SO_4_ were added followed by heating at 70 °C overnight. The mixture was allowed to reach room temperature and the methanol was evaporated and the remaining crude product was dissolved in Et_2_O (15 mL). The organic phase was washed with H_2_O (3 × 10 mL) and brine (2 × 10 mL), dried over Na_2_SO_4_ (anhydr.) and solvent evaporated, resulting in 0.547 g of a yellow oil (92.8% yield). GC-MS (FactorFour vf-23ms) showed a *Z*:*E* ratio of 80:20. ^1^H-NMR(CDCl_3_): 5.4 (m, 2H), 3.67 (s, 3H), 2.3 (m, 4H), 2.03 (q, *J* = 6.5 Hz, 1.6H, *Z*-isomer), 1.96 (q, *J* = 6.5 Hz, 0.4H, *E*-isomer), 1.33–1.21 (m, 8H) and 0.89 (t, *J* = 6.5 Hz, 3H) ppm (no data found in the literature). ^13^C-NMR(CDCl_3_): 134.2, 119.9, 32.3, 31.9, 29.5, 29.3, 27.43, 22.7 and 14.1 ppm; ^13^C-NMR data are in accordance with the literature [68]. Proton NMR showed a 80:20 *Z*:*E* ratio between the diastereomers. Enrichment of the *Z*-isomer on AgNO_3_ (10%) impregnated silica resulted in 63 mg of a 98.6:1.4 *Z*:*E* ratio product according to GC-FID analysis on the vf-5 column as the diastereoisomeric purity was not possible to measure when using ^1^H-NMR.

#### (Z)-4-undecenol

Methyl (*Z*)-4-undecenoate (63 mg, 0.32 mmol) was dissolved in Et_2_O (5 mL) and LiAlH_4_ (2 spatula tips) was added followed by stirring at room temperature for 30 min. HCl (2 M, 2 mL) was added to quench the reaction and the mixture was extracted with Et_2_O (2 × 3 mL), the combined organic layer was dried over MgSO_4_ (anhydr.) and solvent was evaporated. Purification with flash chromatography on SiO_2_ resulted in 37 mg. ^1^H-NMR(CDCl_3_): 5.43–5.32 (m, 2H), 3.67 (m, 2H), 2.16–2.10 (m, 2H), 2.08–2.02 (m, 2H), 1.69–1.60 (m, 2H), 1.39–1.22 (m, 8H) and 0.89 (t, *J* = 6.5 Hz, 3H) ppm. NMR data were similar to that of Kim and Hong [69] and Davis and Carlsson [70]. Diastereomeric purity was checked with GC-FID before the next step.

#### (Z)-4-undecenal

(*Z*)-4-Undecenol (37 mg, 0.22 mmol) in DCM (3 mL) was added to Dess–Martin periodinane (0.140 g, 0.33 mmol) in DCM (0.5 mL). After 50 min, NaOH (2 M, 10 mL) was added to quench the reaction. The two layers were separated and the aqueous phase was extracted with Et_2_O (3 × 10 mL), the combined organic layers were washed with NaOH (2 M, 10 mL), dried over MgSO_4_ (anhydr.) and solvent was evaporated resulting in 30 mg of a yellow oil (81% yield). The crude product was purified with Kugelrohr distillation at boiling point (65–70 °C; 1.6 mbar), resulting in 17 mg. ^1^H-NMR(CDCl_3_): 9.77 (s, 1H), 5.48–5.22 (m, 2H), 2.47 (t, *J* = 7 Hz, 2H), 2.37 (q, *J* = 7 Hz, 2H), 2.04 (q, *J* = 7Hz, 2H), 1.37–1.23 (m, 8H) and 0.88 (t, *J* = 7 Hz, 3H) ppm. ^13^C-NMR(CDCl_3_): 202.1, 131.8, 127.0, 43.9, 31.8, 29.5, 29.0, 27.2, 22.6, 20.1 and 14.1 ppm; both ^1^H- and ^13^C-NMR data were in accordance with published results [71, 72]. Analysis on GC-MS (FactorFour vf-23ms) resulted in a 98.6:1.4 *Z*:*E* ratio, the *E*-isomer could not be detected by ^1^H-NMR.

#### (E)-4-undecenoic acid

A modified version of Virolleaud’s metathesis [73] was used to produce (*E*)-4-undecenoic acid in a 56% yield (87.5% of the *E*-isomer). (*E*)-4-undecenoic acid was esterified under the same conditions as the (*Z*)-acid, without isomerisation of the double bond (according to GC-FID and ^1^H-NMR). The methyl-(*E*)-4-undecenoate was reduced to the alcohol with lithium aluminium hydride in diethyl ether and purified on silver nitrate-impregnated silica gel to obtain a purity of 99.8% of the (*E*)-isomer, which was oxidized with Dess-Martin periodinane in dichloromethane to obtain (*E*)-4-undecenal.

4-Pentenoic acid (0.5 g, 5 mmol) and 1-octene (2.8 g, 25 mmol) were dissolved in DCM (50 mL), Grubbs II catalyst (85 mg, 0.1 mmol) was added and the reaction was refluxed. After 7 h, a second portion of Grubbs II catalyst (85 mg, 0.1 mmol) was added and the reaction refluxed for a further 16 h. The reaction was allowed to reach room temperature and the solvent was evaporated. The obtained crude product was dissolved in Et_2_O (50 mL) and filtered through a short pad of silica gel. The product was purified with flash chromatography by gradient elution (0–100% EtOAc in c-hexane followed by 0–10% EtOH in EtOAC) resulting in 0.52 g of oil (56% yield). ^1^H-NMR(CDCl_3_): 5.51–5.33 (m, 2H), 2.41 (q, *J* = 7 Hz, 2H), 2.32 (q, *J* = 7 Hz, 2H), 2.04 (q, *J* = 6.5 Hz, 0.25 H, *Z*-isomer), 1.97 (q, *J* = 6.5 Hz, 1.75H, *E*-isomer), 1.37–1.22 (m, 9H) and 0.88 (t, *J* = 7.5 Hz, 3H) ppm. The relation between the proton at 2.04 and 1.97 reveals a 87.5:12.5 *E*:*Z* ratio. The isolated product was used in the next step without further purification.

#### Methyl (E)-4-undecenoate

(*E*)-4-undecenoic acid (0.52 g, 2.82 mmol) was dissolved in methanol (25 mL), a catalytic amount H_2_SO_4_ was added and the mixture was refluxed overnight. After evaporation of the solvent, the crude product was dissolved in Et_2_O (10 mL) and washed with H_2_O (20 mL). The aqueous phase was extracted with Et_2_O (2 × 25 mL), the combined organic layer was washed with H_2_O (20 mL) and brine (20 mL), dried over MgSO_4_ (anhydr.) and evaporation of solvent resulted in 0.439 g (78% yield). ^1^H-NMR(CDCl_3_): 5.51–5.33 (m, 2H), 3.67 (s,3H), 2.40–2.27 (m, 4H), 1.96 (q, *J* = 6.5 Hz, 2H), 1.38–1.21 (m, 8H) and 0.88 (t, *J* = 6.5 Hz,3H) ppm. Purification with flash chromatography resulted in 0.401 g (71.7% yield). GC-FID showed the same stereoisomeric ratio as for the acid above.

#### (E)-4-undecen-1-ol

LiAlH_4_ (0.055 g, 1.46 mmol) was added to methyl (*E*)-4-undecenoate (0.145 g, 0.73 mmol) dissolved in Et_2_O (5 mL). After 30 minutes, HCl (2 M, 5 mL) was added to quench the reaction. The acidic water phase was extracted with Et_2_O (3 × 10 mL) and the combined organic layers were dried over MgSO_4_ (anhydr.) and evaporation of solvent resulted in 0.104 g (99% yield). Enrichment of the *E*-isomer with medium pressure liquid chromatography on AgNO_3_ (10% impregnated) silica resulted in 30 mg of a clear oil (>99.8% *E*). ^1^H-NMR(CDCl_3_): 5.43 (m, 2H), 3.65 (m, 2H), 2.08 (q, *J* = 7 Hz, 2H), 1.97 (q, *J* = 7 Hz, 2H), 1.63 (pent, 2H), 1.35–1.21 (m, 9H) and 0.88 (t, *J* = 6.5 Hz, 3H) ppm. ^13^C-NMR(CDCl_3_): 134.4, 131.3, 129.4, 62.6, 32.6, 32.5, 31.7, 29.6, 29.5, 28.9, 28.8, 22.6 and 14.1 ppm. All NMR data were in accordance with previously published data [72].

#### (E)-4-undecenal

Dess-Martin periodinane (0.110 g, 0.26 mmol) was added to (*E*)-4-undecen-1-ol (0.030 g, 0.22 mmol) in DCM (4 mL). NaOH (2 M, 10 mL) was added after 1 h to quench the reaction. The aqueous phase was extracted with Et_2_O (3 × 10 mL) and the combined organic layers were dried over MgSO_4_ (anhydr.); evaporation of the solvent resulted in 30 mg (98% yield). Purification of the crude product with Kugelrohr distillation at 65 °C (2 mbar) resulted in 10 mg of product (33% yield, 97% chemical purity, 3% undecenal). ^1^H-NMR(CDCl_3_): 9.76 (t, *J* = 1.5 Hz, 1H), 5.50–5.36 (m, 2H), 2.48 (d of t, *J* = 7.5, 1.5 Hz, 2H), 2.33 (q, *J* = 7 Hz, 2H), 1.97 (q, *J* = 6.5 Hz, 2H), 1.32–1.19 (m, 8H) and 0.87 (t, *J* = 6.5 Hz, 3H). ^13^C-NMR (CDCl_3_): 202.5, 132.2, 127.6, 43.6, 32.5, 31.7, 29.4, 28.8, 25.2, 22.6 and 14.1 ppm. The NMR data were in accordance with previously published data [72, 74].

### Odour collection and chemical analysis

Groups of 20 flies, 3- to 5-day-old, *D. melanogaster* (Dalby), *D. melanogaster* (Canton-S), or *D. simulans*, unmated females or unmated males (n = 5 for each) were placed in a glass aeration apparatus designed for collection of airborne pheromones (effluvia) [75]. The flies were held in a glass bulb with a narrow open outlet (ø 1 mm), which prevented them from escaping. A charcoal-filtered air flow (100 mL/min) passed over the flies over 75 min. Fly effluvia were collected on the glass surface, breakthrough was monitored by attaching a 10-cm glass capillary (ø 1 mm) onto the outlet. After 75 min, flies were removed and 100 ng of heptadecyl acetate (internal standard) was deposited in the glass bulb, which was then rinsed with 50 μL hexane, and the solvent was concentrated to 10 μL in Francke vials.

Cuticular extracts (n = 5) were obtained by placing 20 *D. melanogaster* females for 5 min in 400 μL hexane containing 100 ng heptadecyl acetate. After 5 min, the extracts were transferred to Francke vials and concentrated to 10 μL before analysis. Fly extracts and volatile collections were stored at –20 °C.

Oxidation of 7,11-HD was analysed by dropping 100 ng of synthetic 7,11-HD into a 1.5-mL glass vial at 19 °C. Vials were rinsed with 10 μL of hexane, which contained 100 ng heptadecyl acetate as an internal standard, after 15, 30, 45, 60 and 75 min (*n* = 3).

Samples were analysed by combined GC-MS (6890 GC and 5975 MS, Agilent technologies Inc., Santa Clara, CA, USA). The samples (2 μL) were injected (injector temperature 225 °C) splitless (30 s) into the fused silica capillary columns (60 m × 0.25 mm) coated with HP-5MS UI (Agilent Technologies Inc., d_f_ = 0.25 μm) or DB-wax (J&W Scientific, Folsom, CA, USA, d_f_ = 0.25 μm), that were temperature-programmed from 30 °C to 225 °C at 8 °C/min. Helium was used as mobile phase at 35 cm/s. The MS operated in scanning mode over m/z range 29–400. Compounds were tentatively identified based on their mass spectra and Kovats retention indices, using custom and NIST (Agilent) libraries, followed by comparison with authentic standards. Each series of GC-MS runs is preceded by blank runs, including solvent, glassware and air filters.

### Behavioural assays

Upwind flight behaviour was observed in a glass wind tunnel (30 × 30 × 100 cm). The flight tunnel was lit diffusely from above, at 13 lux, and the temperature ranged from 22 °C to 24 °C and relative humidity from 38% to 48%, and charcoal filtered air, at a velocity of 0.25 m/s, was produced by a fan (Fischbach GmbH, Neunkirchen, Germany). Compounds were delivered from the centre of the upwind end of the wind tunnel via a piezo-electric micro-sprayer [50, 76]. Forty flies were flown individually to each treatment. ‘Attraction’ was defined as upwind flight, directly from a release tube at the end of the tunnel over 80 cm towards the odour source, followed by landing. Unmated, fed, 4-day-old Dalby wild-type males and females, *D. melanogaster* Zimbabwe strain males and *D. simulans* males were flown towards (*Z*)-4-undecenal (released at 10 ng/min), (*R*)-linalool (10 ng/min) and the blend of (*Z*)-4-undecenal and (*R*)-linalool (10 ng/min, each).

Mated 4-day-old males of the Or69aRNAi/OrcoGal4 line and the respective parental fly lines were used. Since the transgenic fly lines produced fewer offspring, all flies were used, instead of discarding individuals eclosing during the night, which may have mated. Unmated and mated wild-type flies did not show a significant difference in the response rate to 10 ng/min Z4-11Al (50% and 52.5%, *n* = 40).

### Heterologous expression of Or69aA and Or69aB

Or69aA and Or69aB receptors were cloned from antennae of *D. melanogaster* (Dalby) [77]. Briefly, cDNA was generated from RNA extracts of antennae of 100 males and females using standard procedures. Or69a variants were PCR amplified with the following primers: Or69aA_5’: GTCATAGTTGAAACCAGGATGCAGTTGC, Or69aB_5’: ATAATTCAGGACTAGATGCAGTTGGAGG, Or69aAB_3’: TGCACTTTTGCCCTTTTATTTAAGGGAC.

Or69aA and Or69aB were amplified with unique 5’ primers and a common 3’ primer, reflective of genomic structure at this locus. These primers encompass the entire open reading frame of the receptor variants, and are located partially upstream and downstream of the start and stop codons. PCR amplicons were gel-purified and cloned into the pCR8/GW/Topo-TA Gateway entry vector (Thermo-Fisher Scientific, Waltham, MA, USA) according to standard procedure, with vector inserts sequenced to confirm fidelity of Or sequence. Or inserts were subsequently transferred to pUAS.g-HA.attB [78] with LR Clonase II enzyme (Thermo-Fisher Scientific), according to the manufacturer’s protocol; vector inserts were sequenced to confirm fidelity of Or sequence.

Mini-prep purified pUAS.g-HA.attB plasmids with Or69aA or Or69aB insert were delivered to Best Gene Inc. (Chino Hills, CA, USA) for generation of transgenic *D. melanogaster* flies. Using the PhiC31 targeted genomic-integration system [78], vectors with Or69aA or Or69aB were injected into the following fly strain, for integration on the third chromosome M{3xP3-RFP.attP}ZH-86Fb (with M{vas-int.Dm}ZH-2A) (Bloomington *Drosophila* Stock Number: 24749). For expression of single receptor variants in the empty neuron system, Or69a transgenes were crossed into the *Δhalo* background to give genotype w; *Δhalo*/Cyo; UAS-DmelOr69a(A or B), and these flies were crossed to flies with genotype w; *Δhalo*/Cyo; DmelOr22a-Gal4, as described previously [77]. Experimental electrophysiology assays were performed on flies with genotype w; *Δhalo*; UAS-DmelOr69a(A or B)/DmelOr22a-Gal4.

For co-expression of Or69aA and Or69aB in the same empty neurons, a second fly-line with Or69aB was generated with Or69aB present on the X-chromosome. The same UASg-HA.attB:Or69aB plasmid generated previously was injected into the fly strain y,w, P{CaryIP}su(Hw)attP8 (Bloomington *Drosophila* Stock Number: 32233). The Or69aB transgene was crossed into the DmelOr22a-Gal4 line in *Δhalo* background to give genotype UAS-DmelOr69aB; *Δhalo*/Cyo; DmelOr22a-Gal4; these flies were crossed to flies with genotype w; *Δhalo*/Cyo; UAS-DmelOr69aA. Experimental electrophysiology assays were performed on flies with genotype UAS-DmelOr69aB/w; *Δhalo*; UAS-DmelOr69aA/DmelOr22a-Gal4.

Or identity scores were calculated with Clustal Omega Multiple Sequence Alignment webtool, using default parameters [79].

### Conformational analysis

MacroModel version 11.0 (Schrodinger LLC, New York, NY, USA) in the Maestro Version 10.4.017 were used to build, minimize and perform conformational analysis of *Z*4-11Al, (*R*)-carvone, (*S*)-terpineol and (*R*)-linalool, using default settings (OPLS3 as force field, water as the solvent and mixed torsional/low-mode sampling method). The assumed bioactive conformations of the conformationally more flexible compounds, *Z*4-11Al and (*R*)-linalool, were based on the position of the shared functional groups in the conformationally more restricted compounds, (*R*)-carvone and (*S*)-terpineol. The carbonyl and the double bond atoms were kept fixed during minimisation of the proposed bioactive conformation of Z4-11Al; the alcohol functional group and the double bond were kept fixed in (*R*)-linalool. Strain energies, the energy cost for adopting proposed bioactive conformations, were then calculated as the difference between the lowest energy conformations and the assumed bioactive conformation.

### Electrophysiological recordings

SSR were performed as described earlier [23]. Unmated males were restrained in 100-μL pipette tips, with half of the head protruding, the third antennal segment or palps were placed on a glass microscope slide and held by dental wax. For the initial screening, all basiconic, trichoid, coeloconic and intermediate sensilla [36] were localized in *D. melanogaster* (Canton-S strain) males, under a binocular at 1000× magnification. Further recordings were made from small basiconic ab9 sensilla, in *D. melanogaster* (Canton-S and Zimbabwe strains) and in *D. simulans* males, and from large basiconic ab3 sensilla in mutant *D. melanogaster*, where Or69aA and Or69aB were heterologously expressed (see above).

Tungsten electrodes (diameter 0.12 mm, Harvard Apparatus Ltd, Edenbridge, United Kingdom) were electrolytically sharpened with a saturated KNO_3_ solution. The recording electrode was introduced with a DC-3 K micromanipulator equipped with a PM-10 piezo translator (Märzhäuser Wetzler GmbH, Germany) at the base of the sensilla. The reference electrode was inserted into the eye. The signal from OSNs was amplified with a probe (INR-02; Syntech), digitally converted by an IDAC-4-USB (Syntech) interface, and analysed with Autospike software v. 3.4 (Syntech). Neuron activities were recorded during 10 s, starting 2 s before odour stimulation. Neuron responses were calculated from changes in spike frequency, during 500 ms before and after odour stimulation.

Odorants were diluted in redistilled hexane; 10 μg of test compounds in 10 μL hexane were applied to filter paper (1 cm^2^) and kept in Pasteur pipettes. The test panel contained the most active ligands known for Or69a [39] and several aldehydes. Diagnostic compounds for confirmation of sensillum identity were 2-phenyl ethanol (ab9) and 2-heptanone (ab3). Control pipettes contained solvent only. Puffs (2.5 mL, duration 0.5 s) from these pipettes, produced by a stimulus controller (Syntech GmbH, Kirchzarten, Germany), were injected into a charcoal-filtered and humidified airstream (0.65 m/s), which was delivered through a glass tube to the antenna.

For GC-SSR recordings, GC columns and the temperature programmes were the same as for the GC-MS analysis. At the GC effluent, 4 psi of nitrogen was added and split 1:1 in a 3D/2 low dead volume four-way cross (Gerstel, Mühlheim, Germany) between the flame ionization detector and the antenna. Towards the antenna, the GC effluent capillary passed through a Gerstel ODP-2 transfer line that tracked the GC oven temperature, into a glass tube (30 cm × 8 mm ID), where it was mixed with charcoal-filtered, humidified air (20 °C, 50 cm/s).

### Statistical analysis

Generalized linear models with a Bernoulli binomial distribution were used to analyse wind tunnel data. Landing at source and sex were used as the target effects. Post hoc Wald pairwise comparison tests were used to identify differences between treatments. For all the electrophysiological tests, differences in spike activity derived from SSRs were analysed with the Kruskal–Wallis H test followed by pairwise comparisons with the Mann–Whitney U post hoc test. All statistical analyses were carried out using R (R Core Team 2013) and SPSS Version 22 (IBM Corp).

## Abbreviations

7,11-HD: (*Z,Z*)-7,11-heptacosadiene
cVA: cis-vaccenyl acetate
FID: flame ionization detector
GC: gas chromatography
LH: lateral horn
MS: mass spectrometry
Or: olfactory receptor
OSN: olfactory sensory neuron
SSR: single sensillum recording
TeTx: tetanus toxin
*Z*4-11Al: (*Z*)-4-undecenal

## References

[1] Greenfield M (1981). Moth sex pheromones: an evolutionary perspective. Florida Entomol.

[2] Wyatt TD (2010). Pheromones and signature mixtures: defining species-wide signals and variable cues for identity in both invertebrates and vertebrates. J Comp Physiol A.

[3] Wigby S, Slack C, Grönke S, Martinez P, Calboli FC, Chapman T, Partridge L (2011). Insulin signalling regulates remating in female Drosophila. Proc R Soc B.

[4] Gorter JA, Jagadeesh S, Gahr C, Boonekamp JJ, Levine JD, Billeter JC (2016). The nutritional and hedonic value of food modulate sexual receptivity in Drosophila melanogaster females. Sci Rep.

[5] Lihoreau M, Poissonnier LA, Isabel G, Dussutour A (2016). Drosophila females trade off good nutrition with high-quality oviposition sites when choosing foods. J Exper Biol.

[6] Walker SJ, Goldschmidt D, Ribeiro C. Craving for the future: the brain as a nutritional prediction system. Curr Op Insect Sc. 2017. Ahead of print. doi:10.1016/j.cois.2017.07.013. 10.1016/j.cois.2017.07.013 29129289

[7] Endler JA (1992). Signals, signal conditions, and the direction of evolution. Am Naturalist.

[8] Boughman JW (2002). How sensory drive can promote speciation. Tr Ecol Evol.

[9] Gavrilets S (2004). Fitness Landscapes and the Origin of Species.

[10] Bolnick DI, Fitzpatrick BM (2007). Sympatric speciation: models and empirical evidence. Ann Rev Ecol Evol Syst.

[11] Maan ME, Seehausen O (2011). Ecology, sexual selection and speciation. Ecol Lett.

[12] Rosenthal GG (2017). Mate Choice: The Evolution of Sexual Decision Making from Microbes to Humans.

[13] Becher PG, Flick G, Rozpedowska E, Schmidt A, Hagman A, Lebreton S, Larsson MC, Hansson BS, Piskur J, Witzgall P, Bengtsson M (2012). Yeast, not fruit volatiles mediate attraction and development of the fruit fly Drosophila melanogaster. Funct Ecol.

[14] Dweck HK, Ebrahim SA, Kromann S, Bown D, Hillbur Y, Sachse S, Hansson BH, Stensmyr MC (2013). Olfactory preference for egg laying on citrus substrates in Drosophila. Curr Biol.

[15] Laturney M, Billeter JC (2014). Neurogenetics of female reproductive behaviors in Drosophila melanogaster. Adv Genet.

[16] Depetris-Chauvin A, Galagovsky D, Grosjean Y (2015). Chemicals and chemoreceptors: ecologically relevant signals driving behavior in Drosophila. Front Ecol Evol.

[17] Bartelt RJ, Schaner AM, Jackson LL (1985). cis-Vaccenyl acetate as an aggregation pheromone in Drosophila melanogaster. J Chem Ecol.

[18] Grosjean Y, Rytz R, Farine JP, Abuin L, Cortot J, Jefferis GSXE, Benton R (2011). An olfactory receptor for food-derived odours promotes male courtship in Drosophila. Nature.

[19] Kohl J, Ostrovsky AD, Frechter S, Jefferis GSXE (2013). A bidirectional circuit switch reroutes pheromone signals in male and female brains. Cell.

[20] Lebreton S, Trona S, Borrero-Echeverry F, Bilz F, Grabe V, Becher PG, Carlsson MA, Nässel DR, Hansson BS, Sachse S, Witzgall P (2015). Feeding regulates sex pheromone attraction and courtship in Drosophila females. Sci Rep.

[21] Pavlou HJ, Goodwin SF (2013). Courtship behavior in Drosophila melanogaster: towards a 'courtship connectome'. Curr Op Neurobiol.

[22] Clowney EJ, Iguchi S, Bussell JJ, Scheer E, Ruta V (2015). Multimodal chemosensory circuits controlling male courtship in Drosophila. Neuron.

[23] Dweck HK, Ebrahim SA, Thoma M, Mohamed AA, Keesey IW, Trona F, Lavista-Llanos S, Svatos A, Sachse S, Knaden M, Hansson BS (2015). Pheromones mediating copulation and attraction in Drosophila. Proc Natl Acad Sci U S A.

[24] Auer TO, Benton R (2016). Sexual circuitry in Drosophila. Curr Opin Neurobiol.

[25] El-Sayed AM (2017). The Pherobase: Database of Insect Pheromones and Semiochemicals.

[26] Jezovit JA, Levine JD, Schneider J (2017). Phylogeny, environment and sexual communication across the Drosophila genus. J Exp Biol.

[27] Parsons PA (1975). The comparative evolutionary biology of the sibling species, Drosophila melanogaster and D. simulans. Quart Rev Biol.

[28] Coyne JA (1996). Genetics of differences in pheromonal hydrocarbons between Drosophila melanogaster and D. simulans. Genetics.

[29] Billeter JC, Atallah J, Krupp JJ, Millar JG, Levine JD (2009). Specialized cells tag sexual and species identity in Drosophila melanogaster. Nature.

[30] Thistle R, Cameron P, Ghorayshi A, Dennison L, Scott K (2012). Contact chemoreceptors mediate male-male repulsion and male-female attraction during Drosophila courtship. Cell.

[31] Toda H, Zhao X, Dickson BJ (2012). The Drosophila female aphrodisiac pheromone activates pp k23+ sensory neurons to elicit male courtship behavior. Cell Rep.

[32] Everaerts C, Farine JP, Cobb M, Ferveur JF (2010). Drosophila cuticular hydrocarbons revisited: mating status alters cuticular profiles. PLoS One.

[33] Dembeck LM, Böröczky K, Huang W, Schal C, Anholt RR, Mackay TF (2015). Genetic architecture of natural variation in cuticular hydrocarbon composition in Drosophila melanogaster. Elife.

[34] Swedenborg PD, Jones RL (1992). (Z)-4-Tridecenal, a pheromonally active air oxidation product from a series of (Z, Z)-9, 13 dienes in Macrocentrus grandii Goidanich (Hymenoptera: Braconidae. J Chem Ecol.

[35] Cosse AA, Bartelt RJ, Weaver DK, Zilkowski BW (2002). Pheromone components of the wheat stem sawfly: identification, electrophysiology, and field bioassay. J Chem Ecol.

[36] Couto A, Alenius M, Dickson BJ (2005). Molecular, anatomical, and functional organization of the Drosophila olfactory system. Curr Biol.

[37] Syed Z, Ishida Y, Taylor K, Kimbrell DA, Leal WS (2006). Pheromone reception in fruit flies expressing a moth's odorant receptor. Proc Natl Acad Sci U S A.

[38] Lin CC, Prokop-Prigge KA, Preti G, Potter CJ (2015). Food odors trigger Drosophila males to deposit a pheromone that guides aggregation and female oviposition decisions. Elife.

[39] Münch D, Galizia CG (2016). DoOR 2.0 - comprehensive mapping of Drosophila melanogaster odorant responses. Sci Rep.

[40] Robertson HM, Warr CG, Carlson JR (2003). Molecular evolution of the insect chemoreceptor gene superfamily in Drosophila melanogaster. Proc Natl Acad Sci U S A.

[41] Conceicao IC, Aguade M (2008). High incidence of interchromosomal transpositions in the evolutionary history of a subset of Or genes in Drosophila. J Molec Evol.

[42] Dobritsa AA, van der Goes van Naters W, Warr CG, Steinbrecht RA, Carlson JR (2003). Integrating the molecular and cellular basis of odor coding in the Drosophila antenna. Neuron.

[43] Perola E, Charifson PS (2004). Conformational analysis of drug-like molecules bound to proteins: an extensive study of ligand reorganization upon binding. J Medicin Chem.

[44] Ferveur JF, Cobb M, Boukella H, Jallon JM (1996). World-wide variation in Drosophila melanogaster sex pheromone: behavioural effects, genetic bases and potential evolutionary consequences. Genetica.

[45] Dallerac R, Labeur C, Jallon JM, Knipple DC, Roelofs WL, Wicker-Thomas C (2000). A Δ9 desaturase gene with a different substrate specificity is responsible for the cuticular diene hydrocarbon polymorphism in Drosophila melanogaster. Proc Natl Acad Sci U S A.

[46] Yukilevich R, True JR (2008). African morphology, behavior and pheromones underlie incipient sexual isolation between US and Caribbean Drosophila melanogaster. Evolution.

[47] Grillet M, Everaerts C, Houot B, Ritchie MG, Cobb M, Ferveur JF (2012). Incipient speciation in Drosophila melanogaster involves chemical signals. Sci Rep.

[48] Carrau FM, Medina K, Boido E, Farina L, Gaggero C, Dellacassa E, Versini G, Henschke PA (2005). De novo synthesis of monoterpenes by Saccharomyces cerevisiae wine yeasts. FEMS Microbiol Lett.

[49] Hallem EA, Ho MG, Carlson JR (2004). The molecular basis of odor coding in the Drosophila antenna. Cell.

[50] Becher PG, Bengtsson M, Hansson BS, Witzgall P (2010). Flying the fly: long-range flight behavior of Drosophila melanogaster to attractive odors. J Chem Ecol.

[51] Chisholm MG, Jell JA, Cass DM (2003). Characterization of the major odorants found in the peel oil of Citrus reticulata Blanco cv. Clementine using gas chromatography–olfactometry. Flavour Fragrance J.

[52] Tinette S, Zhang L, Robichon A (2004). Cooperation between Drosophila flies in searching behavior. Genes Brain Behav.

[53] Golden S, Dukas R (2014). The value of patch-choice copying in fruit flies. PLoS One.

[54] Lihoreau M, Clarke IM, Buhl J, Sumpter DJ, Simpson SJ (2016). Collective selection of food patches in Drosophila. J Exp Biol.

[55] Shorrocks B, Sevenster JG (1995). Explaining local species diversity. Proc R Soc B.

[56] Krijger CL, Sevenster JG (2001). Higher species diversity explained by stronger spatial aggregation across six neotropical Drosophila communities. Ecol Lett.

[57] Ebrahim SA, Dweck HK, Stökl J, Hofferberth JE, Trona F, Weniger K, Rybak J, Seki Y, Stensmyr MC, Sachse S, Hansson BS (2015). Drosophila avoids parasitoids by sensing their semiochemicals via a dedicated olfactory circuit. PLoS Biol.

[58] Prieto-Godino LL, Rytz R, Cruchet S, Bargeton B, Abuin L, Silbering AF, Ruta V, Dal Peraro M, Benton R (2017). Evolution of acid-sensing olfactory circuits in drosophilids. Neuron.

[59] Ramdya P, Benton R (2010). Evolving olfactory systems on the fly. Tr Genet.

[60] Servedio MR, Van Doorn GS, Kopp M, Frame AM, Nosil P (2011). Magic traits in speciation: 'magic' but not rare?. Tr Ecol Evol.

[61] Thibert-Plante X, Gavrilets S (2013). Evolution of mate choice and the so-called magic traits in ecological speciation. Ecol Lett.

[62] Boughman JW, Svanbäck R (2017). Synergistic selection between ecological niche and mate preference primes diversification. Evolution.

[63] Orr HA, Masly JP, Presgraves DC (2004). Speciation genes. Curr Op Genet Develop.

[64] Nosil P, Schluter D (2011). The genes underlying the process of speciation. Tr Ecol Evol.

[65] Ruebenbauer A, Schlyter F, Hansson BS, Löfstedt C, Larsson MC (2008). Genetic variability and robustness of host odor preference in Drosophila melanogaster. Curr Biol.

[66] Wube AA, Hüfner A, Thomaschitz C, Blunder M, Kollroser M, Bauer R, Bucar F (2011). Design, synthesis and antimycobacterial activities of 1-methyl-2-alkenyl-4(1H)-quinolones. Bioorg Medicinal Chem.

[67] Henderson BS, Larsen BS, Schwab JM (1994). Chemistry and photochemistry attending the inactivation of Escherichia coli beta-hydroxydecanoyl thiol ester dehydrase by an acetylenic diazoketone. J Am Chem Soc.

[68] Bus J, Sies I, Jie MSLK (1976). 13C-NMR of methyl, methylene and carbonyl carbon atoms of methyl alkenoates and alkynoates. Chem Phys Lipids.

[69] Kim SS, Hong YP (2011). The stereospecific synthesis of the rice leaffolder moth sex pheromone components from 1,5-cyclooctadiene. Bull Korean Chem Soc.

[70] Davis TL, Carlsson DA (1989). Synthesis of 7,11-dienes from enol ether and Grignard-reagents under Nickel catalysis: sex pheromones of Drosophila melanogaster. Synthesis.

[71] Wenkert E, Ferreira VF, Michelotti EL, Tingoli M (1985). Synthesis of acyclic, cis olefinic pheromones by way of nickel-catalyzed Grignard reactions. J Org Chem.

[72] Snowden RL, Brauchli R, Linder S (2011). A novel synthesis of γ, δ-unsaturated aldehydes from α-formyl-γ-lactones. Helvetica Chim Acta.

[73] Virolleaud MA, Menant C, Fenet B, Piva O (2006). Total and formal enantioselective synthesis of lyngbic acid and hermitamides A and B. Tetrahedron Lett.

[74] Pearson WH, Hutta DA, Fang WK (2000). Azidomercurations of alkenes: mercury-promoted Schmidt reactions. J Org Chem.

[75] Witzgall P, Frérot B (1989). Pheromone emission by individual females of carnation tortrix, Cacoecimorpha pronubana. J Chem Ecol.

[76] El-Sayed A, Godde J, Arn H (1999). Sprayer for quantitative application of odor stimuli. Environm Entomol.

[77] Gonzalez F, Witzgall P, Walker WB (2016). Protocol for heterologous expression of insect odourant receptors in Drosophila. Front Ecol Evol.

[78] Bischof J, Maeda RK, Hediger M, Karch F, Basler K (2007). An optimized transgenesis system for Drosophila using germ-line-specific phiC31 integrases. Proc Natl Acad Sci U S A.

[79] Sievers F, Wilm A, Dineen D, Gibson TJ, Karplus K, Li W, Lopez R, McWilliam H, Remmert M, Söding J, Thompson JD, Higgins DJ (2011). Fast, scalable generation of high-quality protein multiple sequence alignments using Clustal Omega. Molec Syst Biol.

